# e-Mental Health Intervention Preferences Among Informal Caregivers of Adults Living with Chronic Kidney Disease: Semistructured Interview Study

**DOI:** 10.2196/80962

**Published:** 2026-04-07

**Authors:** Chelsea Coumoundouros, Abla Sami, Paul Farrand, Robbert Sanderman, Louise von Essen, Joanne Woodford

**Affiliations:** 1 CIRCLE – Complex Intervention Research in Health and Care Department of Women’s and Children’s Health Uppsala University Uppsala Sweden; 2 Clinical Education, Development and Research (CEDAR) Psychology University of Exeter Exeter United Kingdom; 3 Department of Health Psychology University Medical Center Groningen University of Groningen Groningen The Netherlands

**Keywords:** chronic kidney disease, e-mental health, informal caregiver, intervention development

## Abstract

**Background:**

Informal caregivers of people living with chronic kidney disease commonly experience mental health difficulties. However, there is currently limited access to scalable psychological interventions such as those using e-mental health platforms. Furthermore, there is an absence of such interventions tailored for this group of caregivers, which may limit acceptability.

**Objective:**

To inform future e-mental health intervention development, this study aimed to explore preferences for e-mental health interventions among informal caregivers of people living with chronic kidney disease.

**Methods:**

A total of 13 informal caregivers of people living with chronic kidney disease participated in semistructured interviews. Eligible informal caregivers lived in the United Kingdom and were recruited via nonprofit organizations and social media advertisements. Semistructured interviews followed a topic guide exploring caregivers’ e-mental health intervention preferences. Data were analyzed using manifest content analysis.

**Results:**

A total of 4 categories and 13 subcategories were generated. “Design preferences” illustrates key intervention design features that were important to caregivers, such as involving caregivers and other interest-holders in the design process. “Content preferences” describes the information caregivers valued in an intervention, such as information about chronic kidney disease and resources to support caregivers. “Support preferences” reflects the characteristics of support caregivers preferred if support was available as part of the intervention, such as peer support and providing support that accommodates individual preferences. Lastly, “Dissemination preferences” describes methods caregivers recommended to promote intervention uptake, such as engaging communication strategies and trusted and accessible sources of information.

**Conclusions:**

Caregivers identified a number of e-mental health intervention design and delivery preferences that should be considered when developing e-mental health interventions for caregivers of people living with chronic kidney disease. To ensure such interventions are acceptable, feasible, and relevant, co-design approaches should be adopted during the intervention development process.

## Introduction

### Background

Chronic kidney disease (CKD) impacts over 800 million people worldwide, is a leading cause of death globally, and significantly impacts quality of life [1-3]. Informal caregivers (caregivers) play a vital role in supporting people living with CKD, providing medical, practical, and emotional support [4-7]. Research suggests caregivers experience worse mental health (eg, symptoms of depression and stress) compared to noncaregivers [8], with caregivers reporting negative impacts, such as high levels of burden, poor quality of life, and mental health difficulties [4,6,9-11]. For example, studies have shown that between 30% and 60% of caregivers of people living with CKD experience symptoms of depression [7,10,11], which is higher compared to the general adult population [12,13]. Consistent with these findings, qualitative research shows that caregivers of people living with end-stage kidney disease experience mental health difficulties due to the caregiving role and could benefit from psychological and emotional support [14]. Ensuring caregivers receive acceptable, feasible, evidence-based mental health support is important not only to ensure caregivers maintain their own well-being, but also because their mental health can also impact the mental health and well-being of the person living with CKD [15,16]. However, caregivers experience multiple barriers to accessing mental health support, such as mental health stigma and negative views of mental health support, limited time due to caregiving responsibilities, and prioritization of the needs of the care recipient [17-19]. All of these barriers may contribute to the low uptake of mental health support among caregivers [20-22].

### e-Mental Health Interventions to Support Caregiver Mental Health

e-Mental health (e-MH) interventions offer one way to provide accessible mental health support to caregivers, given these interventions can be accessed flexibly, without the need for travel or being available for regular appointments [23,24]. The flexibility offered by e-MH interventions can overcome some access barriers experienced by caregivers, such as a lack of time or reliable availability to attend appointments with a mental health professional [17,18]. Additionally, a priority setting initiative highlighted the use of technology as a key research and clinical priority to enhance support for caregivers [25]. e-MH interventions, specifically internet-based cognitive behavioral therapy (CBT) interventions, have been shown to be as effective as comparable in-person interventions among the general population [26], with evidence showing e-MH interventions are also effective for caregivers [27,28].

Although e-MH interventions have been developed for other groups of caregivers (especially caregivers of people living with cancer or dementia) [29], to our knowledge, there are no e-MH interventions tailored to caregivers of people living with CKD, despite interest in this type of intervention [7]. Tailoring e-MH interventions to the intended population has been shown to be important to improve intervention acceptability [30,31]. Additionally, existing mental health interventions for caregivers of people living with CKD identified in the literature are in-person and group-based [32], which may not align with intervention preferences identified by caregivers of people living with CKD [7]. Therefore, the development of an e-MH intervention tailored to the preferences of caregivers of people living with CKD is important to provide accessible mental health support for this group, while ensuring the e-MH intervention is acceptable, feasible, and relevant in practice.

### Approach to e-MH Intervention Development

To develop an e-MH intervention tailored to the preferences of caregivers of people living with CKD, the Medical Research Council framework for the development and evaluation of complex interventions will be followed [33]. This framework emphasizes the importance of considering context and involving interest-holders during the intervention development process as a strategy to enhance the acceptability and future implementation potential of interventions [33]. Our previous research has explored self-help intervention preferences among caregivers of people living with CKD [7] and how interest-holders who may be involved in implementing interventions for this group of caregivers (eg, health and social care professionals, mental health care professionals, and staff at nonprofit organizations) viewed e-MH health interventions for caregivers of people living with CKD [34]. We have also explored caregivers’ current experiences accessing and receiving support from both informal, such as friends and family, and formal, such as health and social care professionals, sources [18]. This study further explores caregivers’ e-MH intervention preferences to provide insights that can be used to inform the development of an e-MH intervention that is acceptable, feasible, and relevant to caregivers of people living with CKD.

### Aim

This study aimed to explore e-MH intervention preferences among informal caregivers of people living with CKD.

## Methods

### Study Design

A qualitative study using semistructured interviews and manifest content analysis [35] was conducted. Results are reported following the SRQR (Standards for Reporting Qualitative Research) checklist [36] (Multimedia Appendix 1). The study sample and data collection procedures are the same as our previous qualitative study exploring caregivers’ experiences of accessing and receiving support [18].

### Ethical Considerations

Ethical approval was obtained from the University of Exeter Psychology Research Ethics Committee (reference 513911) and from the Swedish Ethical Review Authority (dnr 2022-03068-01). Informed consent was obtained from participants prior to data collection. A professional transcription company transcribed audio files, and the resulting transcripts were deidentified by the research team prior to data analysis.

### Sampling

Convenience sampling was used to recruit caregivers using advertisements shared by CKD and/or caregiver nonprofit organizations (eg, via newsletters, email listserv, organization websites, and social media posts), paid and unpaid social media posts on study-specific social media pages, and contacting caregivers who participated in a related study [7] and expressed interest in taking part in an interview. Study advertisements included images of people with a range of genders, ages, and ethnicities to encourage participation of caregivers with diverse backgrounds. Caregivers interested in taking part in an interview contacted the research team via telephone or email. Caregivers were eligible to participate if they were at least 18 years of age, caring for an adult living with CKD, and residing in the United Kingdom, with eligibility confirmed by a research team member via telephone or email prior to the interview.

### Data Collection

Between May 2022 and June 2023, a total of 13 semistructured interviews with a mean length of 63 (range 43-87) minutes were conducted by CC via Microsoft Teams (n=12) or telephone (n=1). The interview guide was partly informed by related qualitative research [17,37,38] and explored topics including support needs, e-MH intervention preferences, and potential barriers and facilitators to intervention use (Multimedia Appendix 2). Before the interview, caregivers were provided with a brief, written description of an e-MH intervention (Multimedia Appendix 2) based on existing low-intensity CBT interventions that have been developed for caregivers experiencing anxiety and/or depression [31,39]. The e-MH intervention was described as a CBT-based online program (eg, app or website) designed to support caregivers experiencing anxiety and/or depression. The intervention content was described as including CBT-based activities and materials (eg, goal setting and behavioral activation), in addition to including information relevant to caregivers of people living with CKD (eg, diet and relevant support services). The intervention was also described as including support from a trained professional, with the option for support to be provided in-person at a convenient location (eg, community center, GP office, or kidney care unit) or via telephone, video-call, email, or SMS text message. The types of support the trained professional would provide were described as including understanding the caregiver’s mental health needs, supporting the caregiver to use the intervention and work through the CBT-based activities, and directing caregivers to additional sources of support (eg, programs within the community, hospital, etc). Interviews were conducted until a rich understanding of intervention preferences was achieved, and no new categories were generated [40]. This was discussed iteratively during data collection by JW and CC.

### Data Analysis

Manifest content analysis with inductive coding was conducted by 2 researchers independently (CC and AS) [35]. All transcripts were read and then coded independently by CC and AS for data familiarization. Coding was undertaken by first creating condensed meaning units, followed by developing codes to describe the condensed meaning units. CC and AS generated categories and subcategories based on their initial coding, resulting in the development of 2 sets of categories and subcategories. CC and AS then held a workshop where they reviewed both sets of categories and subcategories, discussed similarities and differences, and came to a shared understanding of the categories and subcategories that best reflected the data. A single, revised version of categories and subcategories was developed by CC and shared for feedback with AS. This version of categories and subcategories was then shared for feedback with JW and PF, resulting in the development of the final set of categories and subcategories. Trustworthiness was established by having 2 independent researchers code the data prior to discussion, keeping memos throughout analysis, peer examination of categories and subcategories by 2 additional research team members, and inclusion of quotes to support the categories generated during the analysis process [41].

### Researcher Characteristics

Interviews were conducted by CC, who also led data analysis. CC has a public health background and, at the time of the study, was a PhD candidate with experience conducting qualitative research related to caregiver needs and intervention preferences. AS has a community and public health background and has completed training courses in qualitative research. JW has a PhD in Psychology, was the doctoral supervisor of CC, and has extensive experience conducting, teaching, and supervising qualitative research. JW’s research focuses on informal care and mental health. PF is an expert in CBT with extensive qualitative research experience, including research related to kidney disease. Additionally, PF has lived with CKD for over 25 years, during which he has experienced all forms of kidney replacement therapy and is currently on hemodialysis. LvE is a female professor in caring sciences, a psychologist, and specializes in mental health in people affected by somatic disease and their informal caregivers. LvE has extensive experience in teaching, conducting, and supervising qualitative research. RS is a health psychologist and an expert in research on coping with chronic somatic illnesses, interventions (both face-to-face and eHealth) in this area, and has worked extensively with both quantitative and qualitative methods. There are no preexisting relationships between the research team and participants.

## Results

### Sample Characteristics

Caregivers were all female, aged 52 (SD 13) years on average, lived in England (n=12) or Wales (n=1), and had a White (n=11) or South Asian (n=2) background. Caregivers often worked full- or part-time (n=7) while supporting their spouse (n=8), and had been providing care for an average of 7.2 (SD 7.6) years. About half (n=7) of care recipients were receiving kidney replacement therapy (dialysis or transplant). Caregivers provided support with a mean of 10 (SD 6) care-related activities, including cooking, communicating with the medical care team, emotional support, managing symptoms, and running errands. Sample characteristics are summarized in Table 1, with select individual characteristics with caregiver pseudonyms available in Multimedia Appendix 3 to facilitate the interpretation of supporting quotations.

**Table 1:** Summary of caregiver characteristics (n=13).

Characteristic		Value
Age in years, mean (SD; range)		52 (13; 31-67)
Gender (women), n (%)		13 (100)
**Country of residence, n (%)**		
	England	12 (92)
	Wales	1 (8)
**Ethnic background, n (%)**		
	White	11 (85)
	South Asian	2 (15)
**Educational level, n (%)**		
	ISCED^a^ 6 or higher	7 (54)
	ISCED 5	2 (15)
	ISCED 3	2 (15)
	Other	2 (15)
**Employment status, n (%)**		
	Full-time	4 (31)
	Part-time	3 (23)
	Homemaker	2 (15)
	Not working or retired	4 (31)
**Caregiver’s relationship to the care recipient, n (%)**		
	Spouse/partner	8 (62)
	Sibling	3 (23)
	Parent	2 (15)
	Child	1 (8)
**Receipt of kidney replacement therapy, n (%)**		
	Dialysis	3 (23)
	Transplant	4 (31)
	No^b^	6 (46)
Living with care recipient (yes), n (%)		8 (62)
Time in caregiving role (years), mean (SD; range)		7.2 (7.6; 0.25-0.30)
**How well the caregiver is coping, n (%)**		
	Very well	2 (15)
	Well	2 (15)
	Neither well nor not well	6 (46)
	Not well	2 (15)
	Very unwell	1 (8)

^a^ISCED: International Standard Classification of Education.

^b^Individuals not receiving kidney replacement therapy were either not at a stage of disease that required dialysis (n=5) or were receiving conservative kidney management (n=1).

### Qualitative Results

Content analysis generated 4 categories and 13 subcategories reflecting caregivers’ e-MH intervention preferences (Textbox 1).

**Overview of categories and subcategories.**

**Design preferences**

- Considering safety
- Engaging and user-friendly design features
- Involving caregivers and other interest-holders in the design process
- Supporting flexible usage
- Tailoring to cultural and chronic kidney disease (CKD) caregiving contexts

**Content preferences**

- Providing information on CKD and how to support the care recipient
- Supporting emotional well-being

**Support preferences**

- Accommodating individual support preferences
- Knowing support is available
- Ensuring support providers have the right knowledge and skills
- Providing peer support

**Dissemination preferences**

- Engaging communication strategies
- Leveraging trusted and accessible sources

### Design Preferences

#### Overview

This category describes e-MH intervention design features and characteristics that caregivers felt could help ensure an e-MH intervention is considered relevant and could enhance their engagement and trust in the intervention. This category includes the following five subcategories: (1) considering safety, (2) engaging and user-friendly design features, (3) involving caregivers and interest-holders in the design process, (4) supporting flexible usage, and (5) tailoring to cultural and CKD caregiving contexts.

#### Considering Safety

Including design features that would ensure caregivers feel safe using an e-MH intervention was expressed by caregivers in relation to 2 dimensions of safety, that is, psychological safety and data security. Psychological safety related to e-MH interventions that include online discussion forums, where anyone can respond to questions or comments posted by caregivers. Caregivers emphasized the importance of ensuring questions were answered respectfully and the potential need for monitoring in case incorrect information was shared or responses were disrespectful, judgmental, or negative.

I’ve seen things [forums] before where people would genuinely put something there, and you think they’re crying for help, and somebody says, “What are you saying that for? Somebody’s said that before.” And I think that just straight away puts people right off, doesn’t it? And people leave because of those sorts of things.... And if I put something on there and somebody said, “Oh, that’s just stupid. What are you asking that question for?” That would make me think, “well, I’m just not going to bother anymore.”

Emily

Data security related to caregivers’ concerns regarding the security of information that would be shared within the intervention, and ensuring caregivers were informed about how their data were being handled.

You always want to know what’s happening with the information that you give, who uses it, and how.... I think people are open-minded about that [sharing information] when they know.

Olivia

#### Engaging and User-Friendly Design Features

Ensuring e-MH interventions were engaging, not relying solely on written material, and incorporated opportunities for interaction was valued by caregivers. Opportunities for interaction that could be included within an e-MH intervention suggested by caregivers included creative activities, such as art-based activities and opportunities to interact with people, including other caregivers (eg, an online forum where caregivers could ask questions) and health care professionals (eg, a live session or talk with health care professionals, including time for caregivers to ask questions).

But I think you can’t beat human contact. And if we’ve learned anything through the pandemic, it’s that people need people.

Olivia

Ensuring the intervention was user-friendly was viewed as another important element to support caregiver engagement. Key design considerations suggested by caregivers to ensure the design of a user-friendly intervention included offering the intervention as an app to allow caregivers to access the intervention quickly on their mobile phone and presenting information in an “easy to digest” (Freya) way using easy-to-understand language. Designing an intervention to meet the needs of caregivers with different levels of digital literacy was also emphasized as important to ensure the intervention is “straightforward” (Emily) to use and does not “presume that everybody’s quite techy” (Emily).

#### Involving Caregivers and Other Interest-Holders in the Design Process

Involvement of people with lived experience, such as caregivers, people living with CKD, and other interest-holders with relevant expertise (eg, kidney care providers), during intervention development was recommended by caregivers as a key strategy to enhance their trust and positive views of an e-MH intervention.

I think when you’re setting it [the e-MH intervention] up, when you’re developing it, get people like me and people you’ve interviewed to test drive it for you. Because end users will tell you how it’s working, [and if] it’s meeting their need.

Zainab

Sustained involvement of people with lived experience and relevant expertise, such as having a project advisory group including public contributors and other interest-holders during initial testing and later intervention implementation, was also suggested as a strategy to ensure their perspectives continue to inform refinement of the intervention design. Such involvement was also perceived as important to demonstrate that the research and intervention development team is responsive to caregivers’ preferences.

#### Supporting Flexible Usage

The online format was perceived as making e-MH interventions flexible and thus feasible for caregivers to access and use. Caregivers expressed the importance of the ability to choose when and where to access the intervention and how long to engage with it at one time. This flexibility was perceived as supporting caregivers’ ability to balance their caregiving responsibilities and needs (eg, the need to limit contact with others due to COVID-19, with intervention use).

Also, if it’s online, it means that we can do it whilst we are cooking, it means we can do it after work, or whatever.

Priya

Additionally, caregivers wanted to be able to access information in the intervention based on their own needs rather than needing to work through all the content in a set order. The intervention’s online format was viewed as meeting this preference for flexible usage.

#### Tailoring to Cultural and CKD Caregiving Contexts

An e-MH intervention specifically for caregivers was valued as a way to provide a tailored support resource that would align with caregivers’ needs and context. Tailoring the intervention to caregivers of people living with CKD was seen as important to enhance the perceived relevance of the intervention, which could encourage caregiver engagement. Caregivers expressed preferences for e-MH interventions tailored to recognize the caregiving experience. For example, addressing the “practical side and the emotional side” (Zainab) of caregiving, and including information and language relevant to caring for someone living with CKD.

I would want, as you mentioned, some intervention where it is tailored towards people that are caring for people with kidney disease and renal related issues. So, things like [including] useful terminology, using the language that kidney patients use, and carers use. I think that would be really helpful because it would set the tone, it would feel like the right environment.

Priya

Two caregivers had South Asian backgrounds, and they also mentioned the importance of cultural tailoring and cultural sensitivity when developing the e-MH intervention. For example, being aware of various religious holidays when scheduling live events or support sessions.

### Content Preferences

#### Overview

This category describes the information and resources caregivers felt should be included within an e-MH intervention. This category has two subcategories: (1) providing information on CKD and how to support the care recipient, and (2) supporting emotional well-being.

#### Providing Information on CKD and How to Support the Care Recipient

Caregivers were interested in receiving information about CKD, including the stages, treatments (eg, dialysis, transplants, and side effects), diet, and information about living with someone who is living with a transplant and is immunosuppressed. Information was desired not only to understand the care recipient’s current CKD stage but also to prepare for the progression of CKD to other stages and to understand future care and treatment needs.

I think for me, it [intervention content] would be what the future would be like, about dialysis, transplant, and what life would be like. Just so you can prepare yourself emotionally—this is what my life is going to be like.

Freya

Caregivers also wanted information that would help them to support the care recipient. This included information on how to provide them with emotional support so they “feel better in themselves as well” (Sarah), motivate them to do things to support their health and well-being (eg, attend medical appointments), and communicate effectively with the care recipient’s care team to ensure they receive optimal care. Preferences for the intervention to include information about CKD were also connected to caregivers’ desire for an intervention that would not only support them as caregivers, but also help them understand what the care recipient was experiencing and how to help monitor their health.

#### Supporting Emotional Well-Being

Caregivers expressed a need for resources offering practical tips and strategies they could use to practice self-care and manage the different emotions they had to cope with while caring, such as coping with uncertainty and anxiety or feeling low:

I think ways of distracting yourself and ways of being kind to yourself really, suggestions of something you can do that’s mindfulness, that takes your mind away from the whole caring situation.

Sarah

In addition to resources supporting their emotional well-being, caregivers were interested in strategies to help them balance different roles and responsibilities while identifying when to reach out for support. An e-MH intervention with practical, concrete activities caregivers could use in their daily routines, rather than just providing information, was viewed as something that would increase the perceived value of the intervention.

### Support Preferences

#### Overview

This category describes characteristics of both the type of support that should be available within an e-MH intervention and the characteristics of individuals who would support the intervention. This category has the following four subcategories: (1) accommodating individual support preferences, (2) knowing support is available, (3) ensuring support providers have the right knowledge and skills, and (4) providing peer support.

#### Accommodating Individual Support Preferences

Support that could be customized to accommodate caregivers’ individual preferences was preferred to ensure support would be accessible and acceptable to caregivers. Caregivers expressed mixed preferences regarding the support format, with in-person, written, telephone, and video-call support being mentioned. Flexibility to select the support format and change it based on needs and preferences was expressed, with Kate explaining that “if somebody doesn’t want to physically talk, there is that option of just sending something [written].” Support should also be responsive to the type of support the caregiver wanted, for example, whether a caregiver wants emotional support or informational support (ie, to be directed to relevant resources and information). In-person support was viewed as an important way to connect with the support provider and facilitate relationship building and trust. However, in-person support sessions were not always viewed as feasible due to a lack of time and the potential burden of having to travel to the session. In addition to format preferences, the ability to select a support provider based on the provider’s characteristics, such as gender or cultural background, was viewed as a way to further meet caregivers’ preferences and help caregivers feel more comfortable seeking support:

I think having a mix of genders, sexualities [among support providers]—I think it’s important because men might feel comfortable talking to men, women might feel comfortable [talking to women], or it might be the opposite. I think having access to both would really help.

Priya

#### Knowing Support Is Available

Caregivers expressed a preference for support to be available as part of an e-MH intervention, given it would be “nice to know that there is support there [in the intervention]” (Sarah). Caregivers felt that support providers should take active steps to support them by reaching out periodically rather than relying on the caregiver to initiate contact with the support provider. Support is needed to address caregivers’ emotional support needs by building a relationship with the caregiver and providing encouragement:

And I think some, just some positive reinforcement along the way that you’re doing the best that you can in this moment.

Olivia

Additionally, practical support to assist caregivers in using the intervention, such as technical support, understanding and applying intervention content in practice, and checking on caregivers’ progress to motivate intervention usage, was important:

When I come across something I don’t understand or I don’t feel confident about, that’s when I need support.

Holly

#### Ensuring Support Providers Have the Right Knowledge and Skills

Caregivers felt support providers would need to have a range of skills to meet caregivers’ support needs, including cultural sensitivity, emotional support skills (eg, empathy and active listening), knowledge of CKD, knowledge of the intervention (eg, navigating intervention content), and an understanding of the caregiving experience:

Maybe somebody who’s had some training on counselling or somebody who’s empathetic. It [the support provider] needs to be somebody who can try and understand where you’re at and where you are coming from.

Rebecca

It was acknowledged that a single support provider may not have the full range of knowledge and skills to meet all support needs. It was suggested that support providers could vary based on the type of support needed. For example, a support provider could be available for CKD medical information needs, and potentially a different provider for emotional support needs.

#### Providing Peer Support

Caregivers wanted an e-MH intervention to include opportunities to connect with other caregivers with shared characteristics, such as a similar age range or lived-experience (eg, caring for a young family and someone living with CKD). Shared lived experience was viewed as important to foster understanding and provide validation throughout the caregiving journey. Peer support was seen as a way to receive advice from other caregivers, including caregivers with more experience who may be able to provide insights on caring for people at different stages of CKD:

I think what I would really love is to meet somebody to say ‘I was in that situation’.... It’s all very well reading about it [CKD], but you still, you’ve got all those questions to ask. In terms of how much medicine is going to be in the house or how many hospital visits will there be and how will it impact on the whole family and things like that. So yes, first-hand experience, I think would be very helpful.

Sarah

However, depending on the format of peer support, guidelines and monitoring were perceived to be needed to ensure peer support provided is nonjudgmental and confidential.

### Dissemination Preferences

#### Overview

This category describes key elements that should be considered when sharing information about the e-MH intervention with caregivers. This category includes 2 subcategories: (1) engaging communication strategies and (2) leveraging trusted and accessible sources.

#### Engaging Communication Strategies

Caregivers expressed that communication strategies used to promote the e-MH intervention would need to create interest and excitement in the intervention to motivate uptake. They suggested implementing communication strategies using short, easy-to-understand language that clearly expresses the purpose of the intervention so caregivers can quickly understand who should use the intervention and how it will meet their needs:

You can get bogged down, especially on our journey, with lots of words that you either don’t understand or they’re not in your day-to-day vocabulary. So, you would spend more time trying to Google what it meant. So, if something is bright, if it’s clear, if it’s short, if it’s concise, those are the things that engage me.

Kate

Caregivers suggested that communication strategies should leverage factors they identified as motivating their uptake of an e-MH intervention, including an interest and desire to learn, the opportunity to try a new intervention, and evidence (eg, testimonials that other caregivers benefited from the intervention).

And knowing that you are part of a...pilot scheme, it’s quite exciting, it’s something new, it’s something exciting, you know. And it’s something that you want to be actively participating in.

Claire

#### Leveraging Trusted and Accessible Sources

Caregivers expressed that an e-MH intervention should be endorsed by a trusted source to enhance their confidence that the intervention and the information provided within it would be trustworthy. A variety of sources were considered trustworthy, including the kidney care unit, nonprofit CKD and caregiver organizations, and other caregivers:

I think using the kidney charities would be a really good way [to promote the intervention]. If they promote it, then you’d feel this is endorsed by someone I trust to have good information. I think if you just saw a random advert you’d think, ‘is this actually legit?’

Freya

In addition to considering the trustworthiness of the intervention source, the accessibility and reach of the intervention source were other important considerations regarding who should disseminate information about the e-MH intervention. Not all potential intervention sources were considered accessible to all caregivers; for example, some caregivers had limited contact with the kidney care unit due to living far from the unit or not always attending appointments with the person they were caring for. Additionally, some caregivers felt that health care professionals can be overburdened and focused on the person living with CKD, and therefore, may not be the best source of information about interventions for caregivers.

## Discussion

### Principal Results

#### Overview

This study explored the preferences of caregivers of people living with CKD that require consideration to inform the development of an acceptable and relevant e-MH intervention for this group of caregivers. Study findings illustrated preferences for an e-MH intervention tailored to the context of caregivers in relation to intervention design, intervention content, support options, and dissemination approaches. Findings align with the Medical Research Council framework for the development and evaluation of complex health care interventions, which highlights the importance of considering context (eg, the caregivers’ background and their caregiving situation) as a core component of the intervention development process [33].

#### e-MH Intervention Preferences

Many preferences identified in this study align with intervention preferences identified in other caregiving literature, such as the need for practical, disease-specific content, the provision of empathetic and knowledgeable support from a trained provider, and flexible options for how to use and engage with the intervention [7,42-44]. Although caregivers commonly report wanting information about the care recipient’s illness [7,42], this study identified an interest in not only information related to the care recipient’s current stage of disease, but also advanced stages as a way to prepare for the future. In contrast, a study focused on caregivers of people living with cancer found a preference for information specific to the care recipient’s current stage of disease [42]. This difference may relate to the progressive nature of the CKD trajectory and the increase in care needs as the disease progresses to more advanced stages [45]. Additionally, other work suggests that seeking and obtaining information is important to support self-management of CKD by people living with CKD and their caregivers and that unmet information needs impact caregivers’ mental health [46,47]. This highlights the importance of addressing caregiver information and education needs within an e-MH intervention.

Although the preferences identified in this study align with other research, it is important to recognize that this study only captured the preferences of women caregivers. Many e-MH intervention preferences reported by men align with the findings of this study, with men valuing easy-to-use e-MH interventions with flexible engagement options (eg, content available in multiple formats or the option to receive support from a trained provider) [48]. However, some e-MH intervention preferences may vary by gender; for example, one study found that men preferred e-MH interventions with a video-game format more than women [49]. Potential gender differences in e-MH intervention preferences highlight the importance of including caregivers with diverse gender identities in the intervention development process to enhance intervention acceptability.

#### Contrasting Caregiver and Other Interest-Holder e-MH Intervention Preferences

To enhance the future implementation potential of an e-MH intervention for caregivers, the perspective of other interest-holders who would be involved in intervention delivery and dissemination is vital [33]. Our related research exploring potential implementers’ (ie, professionals working in kidney care, mental health care, and nonprofit organizations) perspectives of an e-MH intervention for caregivers of people living with CKD [34] identified a number of similarities to the caregiver perspectives explored in this study. Potential implementers also viewed e-MH interventions as a flexible means of providing mental health support that could fit within caregivers’ caring responsibilities, and that tailoring of intervention content and availability of support within the intervention were important design features [34]. However, they raised concerns regarding e-MH intervention access and digital exclusion [34]. Although caregivers in this study acknowledged designing an intervention to suit different digital literacy levels was important, they did not express similar concerns regarding digital exclusion. Since interest-holder views of e-MH intervention design and caregivers’ ability to use the intervention are important implementation determinants [50], concerns regarding digital exclusion should be addressed as the intervention development process continues. Strategies to address these concerns and support intervention access for caregivers with lower digital literacy should include training to show caregivers how to use the intervention [41,42] and providing a nondigital version [34]. Continued engagement of caregivers and interest-holders in the co-design of the e-MH intervention will be needed to ensure the intervention is user-friendly and meets the preferences of relevant user groups [51].

#### Trust in e-MH Interventions

Trust was identified by caregivers as an important design element of e-MH interventions in relation to the credibility of the intervention and its content, trust in the organization disseminating or endorsing the intervention, and data security. Trust in e-MH interventions has only been minimally explored in the caregiving literature [29,34,52,53]; trust is an important element of eHealth intervention design [54]. For example, persuasive system design, an approach to designing digital interventions and technologies to change end users’ attitudes or behaviors, highlights technology credibility as an important intervention feature that can be built through strategies such as highlighting the expertise of the intervention developers and the intervention’s evidence base [55]. Another study suggests that aligning intervention features with user preferences is another way to build trust in e-MH interventions [54]. Applying this to the user preferences identified in this study, designing an e-MH intervention with multiple support format options that caregivers can choose from, such as written or in-person support, and having a gender and culturally diverse group of support providers such that caregivers can choose a support provider they feel most comfortable working with, could help foster trust of the e-MH intervention [54]. Additional strategies to enhance trust based on our findings and related research include leveraging trusted sources, such as health care providers and established nonprofit organizations, to disseminate information about the intervention, and involving caregivers and other interest-holders with relevant expertise in intervention development [34]. Incorporating strategies to build trust and credibility in the development of e-MH interventions will be important to support intervention adoption [54,56].

### Implementation Considerations

An e-MH intervention for caregivers could fit well within a collaborative care model [57,58], whereby a central contact could link caregivers to health and social care professionals who can address their individual support needs. These could include facilitating a connection with the kidney health care team for support with medical information needs or a connection to a mental health professional for support with mental health needs. This approach to the provision of support aligns with the preference expressed in this study for support to be provided by providers with the appropriate competencies based on the caregivers’ specific support needs. Collaborative care models have been shown to benefit caregivers, for example, improving caregivers’ ability to cope and providing reassurance through the provision of accessible and acceptable support [59,60]. A collaborative care model to support caregivers of people living with CKD could be supported by the Assistant Wellbeing Practitioner (Kidney) role that has been proposed to be integrated within kidney units to provide mental health support to people living with CKD and their caregivers while working with multidisciplinary kidney care teams [61]. Competencies for the Assistant Wellbeing Practitioner (Kidney), including having knowledge related to mental health and CKD, and using psychologically informed approaches to support caregivers of people living with CKD, such as supporting caregiver use of digital interventions [61], align with the support preferences expressed by caregivers in this study. However, this role has not yet been implemented throughout the health care system.

Alternatively, nonprofit, community organizations have also been endorsed by caregivers [7] as an alternative implementation context for an e-MH intervention for caregivers of people living with CKD. Interest-holders working within community organizations also viewed an e-MH intervention for caregivers as aligning with the mission of their organization [34]. Implementation of e-MH interventions for caregivers within community organizations could align with the dissemination preferences expressed in this study, which highlighted that health care professionals can be viewed as less accessible to caregivers, given that not all interact with the care recipient’s care team. Another factor that may facilitate implementation of an e-MH intervention for caregivers within community organizations is the preference for intervention support to be provided by peers (ie, people with lived experience of caring for someone living with CKD) and the high value caregivers place on the knowledge gained from lived experience [18,62]. Shared lived experience can support relationship building, which is an important common factor in mental health interventions [63]. There is also evidence that e-MH interventions supported by peers are as effective as e-MH interventions supported by mental health care professionals [64]. Leveraging peers to provide support as part of an e-MH intervention for caregivers may also enhance the feasibility of implementing an e-MH intervention, given existing capacity challenges within the mental health care system [65] and the lack of psychosocial care professionals within the kidney care sector [66]. Therefore, community organizations should also be considered as a possible implementation setting for an e-MH intervention for caregivers.

### Limitations

The perspectives of some potentially important subgroups of caregivers may not have been captured in this study, including the perspective of men caring for someone living with CKD, and caregivers from a wider range of cultural backgrounds. Caregiver mental health status was not assessed in this study; therefore, the preferences identified may not reflect the preferences of caregivers experiencing common mental health difficulties, such as anxiety or depression, who are the intended users of the e-MH intervention. However, the perspectives of caregivers who were coping well and not well were included in this work. Future studies may want to focus on recruiting caregivers reporting current or past experiences of common mental health difficulties to further inform e-MH intervention development. Caregivers were recruited using advertisements on various online platforms. Therefore, the views of caregivers with lower digital literacy may not have been captured in this study. As e-MH intervention development continues, strategies to engage caregivers with low digital literacy (eg, recruitment via newspaper or printed advertisements and in-person data collection opportunities) should be explored. Finally, caregivers’ e-MH intervention preferences were obtained based on a text-based description of e-MH interventions, which could be interpreted differently based on caregivers’ previous experiences and interactions with e-MH or other digital health interventions. Future work developing the e-MH intervention for caregivers of people living with CKD should continue to engage caregivers, leveraging recruitment strategies to enhance participant diversity [67], in the co-design process as tangible intervention prototypes are developed to ensure the intervention continues to align with caregivers’ needs and preferences.

### Conclusions

This study identified a number of preferences regarding e-MH interventions among caregivers of people living with CKD that can be used to inform future intervention development. Ensuring interventions are tailored toward caregivers’ context, include content that meets caregivers’ information needs and preferences, have flexible access and support options to accommodate caregivers’ caring responsibilities and support needs, and are disseminated using engaging messaging via trusted sources were key design considerations. Future work should use the results of this study to codevelop e-MH intervention prototypes to continue the intervention development process, ensuring continued collaboration and feedback from caregivers and other interest-holders with relevant expertise.

## Appendix

Multimedia Appendix 1 SRQR checklist.

Multimedia Appendix 2 Caregiver interview guide.

Multimedia Appendix 3 Individual caregiver characteristics.

## Abbreviations

CBT: cognitive behavioral therapy
CKD: chronic kidney disease
e-MH: e-mental health
SRQR: Standards for Reporting Qualitative Research

## Data Availability

The datasets generated and/or analyzed during this study are not publicly available to maintain participants’ privacy and confidentiality, but may be provided upon reasonable request from JW.
