# Supplementary material for: e-Mental Health Intervention Preferences Among Informal Caregivers of Adults Living with Chronic Kidney Disease: Semistructured Interview Study
Source: JMIR Hum Factors. 2026 Apr 7;13:e80962. doi: 10.2196/80962 (PMC13100580; doi:10.2196/80962)
Supplement: Multimedia Appendix 2 [file humanfactors_v13i1e80962_app2.docx]

**Multimedia Appendix: Intervention description and interview guide**

**A. Intervention description**

Below is a brief description of what an online emotional support programme for family members, partners, and friends of people living with kidney conditions might look like.

We would like to develop an online emotional support programme specifically designed for family members, partners, and friends of people living with kidney conditions. Online emotional support programmes can include things like websites and smartphone applications.

We would like to hear what you think about the idea of providing emotional support in an online programme.

***What might the emotional support programme look like?***


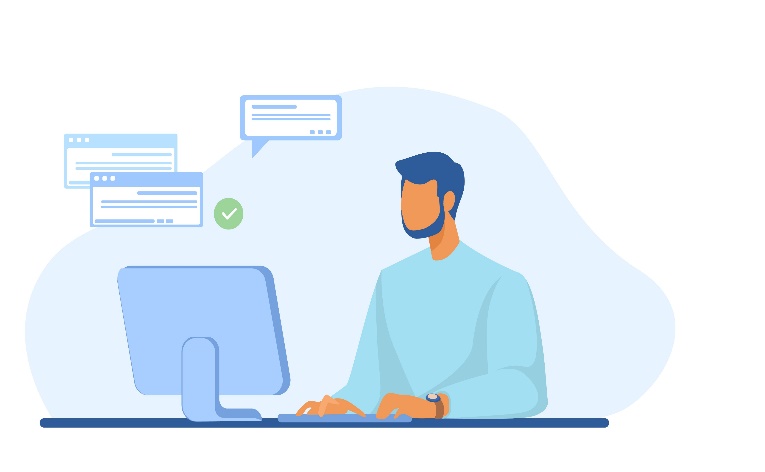
The programme will be designed to help family members, partners, and friends of people living with kidney conditions with their emotional wellbeing, for example feeling low, down, anxious, worried, or stressed.

The programme will be based on evidence-based techniques from cognitive behavioural therapy.

The programme will also include other important information that family members, partners, and friends of people living with kidney conditions might find helpful, for example, communicating with the person you are supporting, returning to work, diet and exercise, sexual relations and intimacy, and information about relevant support services.

The programme will be designed to last for about 6 weeks and will include weekly homework activities (for example, thinking about what is important to you, planning tasks, setting goals for yourself).

The programme will be delivered online using the internet. For example, accessed via a website or a smartphone application. The programme can consist of text, illustrations, film, and audio files. Typically online emotional support programmes include different modules or sections, for example, with specific techniques from cognitive behavioural therapy, and information that might be helpful for family members, partners, and friends of people living with kidney conditions.

People using the programme will be supported by someone trained in how to provide support and guidance to people using online emotional support programmes. Support will be received on a regular basis (for example, weekly) and can be provided face-to-face, over the telephone, via video-conference, SMS, or email.


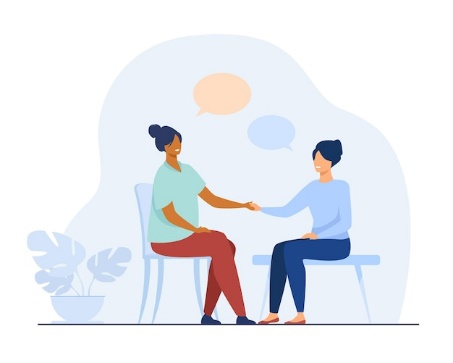

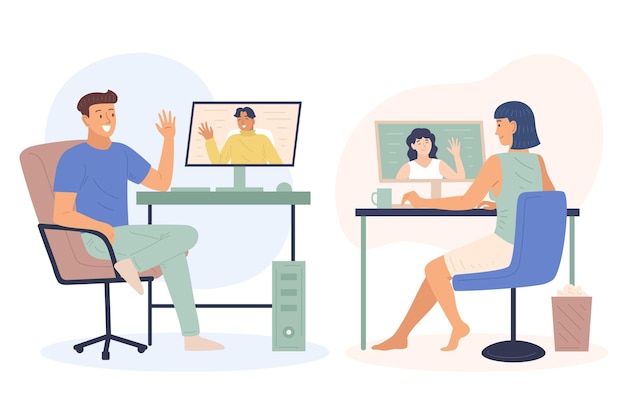


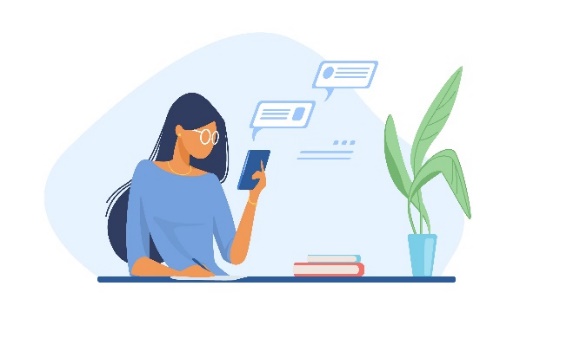


The person providing support and guidance will be trained. However, they are not required to have a core physical health or mental health professional qualification.

The person providing support and guidance may be involved in activities such as:

- Understanding what type of emotional support the person using the programme needs
- Supporting/guiding people to use the cognitive behavioural therap- based techniques within the online emotional support programme
- Signposting people using the programme to other sources of support (including health and social care professionals and other sources of community based support)

If support and guidance is provided face-to-face, this could be in a variety of different locations, for example, in a renal/satellite unit, at the hospital, at a GP practice, within a mental health service, or within a community setting.

**B. Interview Guide for Caregivers**

Welcome the caregiver to the interview [Important: ensure participants are reminded of the purpose of the interview and re-affirm consent].

1. Can you tell me a little about your caring situation?
   1. PROMPTS: Who do you care for? What kidney condition do they have? What stage are they at? What type of treatment are they receiving? How long have you cared for them? What type of things do you help them with (e.g. practical support, emotional support, decision making etc)?

**Needs**

1. What type of support would you like to receive to help you in your caring role?
   1. PROMPTS: Information about [friend, family member, partner’s] condition or support resources? Financial support? Practical help with providing care? Help balancing work and providing care? Emotional support?
   2. PROMPT (if response is focused on support received): Is there a type of support that you wish you would have had, but have not received?

**Sources of support**

1. What type of support have you received from healthcare professionals in relation to your role helping [friend, family member, partner]?
   1. PROMPTS: What were your impressions of these healthcare professionals (e.g. trustworthy/reliable, approachable)? What went well in your interactions with them? What did not go so well in your interactions with them?
   2. What type of support did they provide you with (e.g. information, practical support, emotional support, referral to other resources)? How helpful was the support you received from them?
   3. PROMPT: What support from healthcare professionals have you received in relation to your own emotional wellbeing?
2. What type of support have you received from community organisations or charities (e.g. Kidney Care UK, National Kidney Federation, Kidney Patient Associations, Carers UK) in relation to your role helping [friend, family member, partner]?
   1. PROMPTS: What were your impressions of these community organisations (e.g. trustworthy/reliable, approachable)? What went well in your interactions with them? What did not go so well in your interactions with them?
   2. What type of support did they provide you with (e.g. information, practical support, emotional support, referral to other resources)? How helpful was the support you received from them?
   3. PROMPT: What support from community organisations or charities have you received in relation to your own emotional wellbeing?
3. What type of support have you received from your family, friends, neighbours, and colleagues in relation to your role helping [friend, family member, partner]?
   1. What type of support did they provide you with (e.g. information, practical support, emotional support, referral to other resources)? How helpful was the support you received from them?
   2. PROMPT: What support from family, friends, neighbours, and colleagues have you received in relation to your own emotional wellbeing?
   3. Sometimes when people start helping someone with a chronic kidney condition, they find that their social network, e.g., the network of people you interact with and have relationships with, can change. In what ways has your social network changed since you started helping someone with a kidney condition?
      1. PROMPTS: What happened with the people you already knew (did you see them more or less, how if your relationship with them now)? What new relationships have you made?

**Barriers and facilitators to accessing support**

1. What factors have made it difficult/stopped you from getting support?
   1. PROMPTS: lack of time, hard to make time for yourself, don’t know where to go, uncomfortable talking to people about need for support?
2. What has helped you/made it easier to get support?
   1. PROMPTS: do you know where to go if you need support? Do you feel you have someone you can speak to if you need support? Are you comfortable asking for support?

**Views on e-mental health**

Next I want to ask you a few questions about online emotional support programmes. As part of this project, we asked you to read through some brief material describing what online emotional support programmes are. [Summarize material before moving to next question].

1. What is your experience of using online programmes such as websites and smartphone applications in connection with your emotional wellbeing?
   1. Can you give me examples of when you have used an online programme in connection with your emotional wellbeing?
   2. If not, what are your thoughts about receiving an emotional support programme using digital solutions?

9. What would an online emotional support programme need to include to meet your needs and preferences?

a. PROMPTS: What topics should be included? Who would you like to support the programme? How would you like to be supported e.g., face-to-face, telephone, video-conference, email? How should the programme be made available (e.g., through the kidney unit, a community organization, general practitioner)?

10. What opportunities or benefits do you see for an online emotional support programme to be successful in supporting your emotional health?

a. PROMPT: What would motivate you to use an online emotional support programme? Helpful in overcoming practical barriers? Stigma. Time.

11. What obstacles do you see for an online emotional support programme to be successful?

a. PROMPTS: Safety, security, trust, burden of use, perceived effectiveness.

12. Is there anything else you would like to share about your experiences accessing support, your needs, or about online emotional support programmes.
