# Supplementary material for: e-Mental Health Intervention Preferences Among Informal Caregivers of Adults Living with Chronic Kidney Disease: Semistructured Interview Study
Source: JMIR Hum Factors. 2026 Apr 7;13:e80962. doi: 10.2196/80962 (PMC13100580; doi:10.2196/80962)
Supplement: Multimedia Appendix 3 [file humanfactors_v13i1e80962_app3.docx]

**Multimedia Appendix: Individual caregiver characteristics**

| **Name** | **Age** | **Relationship to the care recipient(s)** | **Currently living with care recipient** | **Receipt of renal replacement therapy** | **Length of time caring** | **How well they were coping** |
| --- | --- | --- | --- | --- | --- | --- |
| Anna | 60-69 | Spouse/Partner | No^b^ | Yes, dialysis | 6 years | Neither well nor not well |
| Chloe | 40-49 | Spouse/Partner | Yes | Yes, received transplant | 11 years | Neither well nor not well |
| Claire | 60-69 | Spouse/Partner | Yes | No^d^ | 9 years | Very well |
| Emily | 60-69 | Spouse/Partner | Yes | Yes, dialysis^d^ | 1 year 7 months | Not well |
| Freya | 30-39 | Spouse/Partner | Yes | No | 5 years | Well |
| Kate | 40-49 | Spouse/Partner & Parent^a^ | Yes | No | 2 years 6 months | Very well |
| Olivia | 40-49 | Spouse/Partner | Yes | Yes, received transplant | 4 years 6 months | Neither well nor not well |
| Sarah | 40-49 | Spouse/Partner | Yes | No | 3 months | Very unwell |
| Priya | 30-39 | Sibling^a^ | No | Yes, received transplant | 30 years | Well |
| Rebecca | 60-69 | Sibling | No | No | 10 years 6 months | Neither well nor not well |
| Sofia | 40-49 | Sibling | No^c^ | Yes, dialysis | 5 years | Not well |
| Holly | 60-69 | Child | Yes | No | 5 years 6 months | Neither well nor not well |
| Zainab | 50-59 | Parent | No | Yes, received transplant | 2 years 9 months | Neither well nor not well |

Note: Pseudonyms and age ranges are used to protect participant confidentiality

^a^Kate and Priya both care for two people living with CKD.

^b^Anna’s spouse typically lived with her, however, at the time of the interview he was hospitalised.

^c^Sofia provided long-distance care for her brother who lives in another country.

^d^Claire and Emily were caring for someone with CKD related to having kidney cancer.
